# Potential Cooperations between Odorant-Binding Proteins of the Scarab Beetle *Holotrichia oblita* Faldermann (Coleoptera: Scarabaeidae)

**DOI:** 10.1371/journal.pone.0084795

**Published:** 2013-12-23

**Authors:** Bing Wang, Li Guan, Tao Zhong, Kebin Li, Jiao Yin, Yazhong Cao

**Affiliations:** State Key Laboratory for Biology of Plant Diseases and Insect Pests, Institute of Plant Protection, Chinese Academy of Agricultural Sciences, Beijing, People’s Republic of China; Indian Institute of Science, India

## Abstract

It was previously thought that the odorant binding proteins (OBPs) in the sensillum lymph might serve as carriers, which could carry lipophilic odorant molecules to olfactory receptors. In this study, two novel OBP genes of the scarab beetle *Holotrichia oblita* were screened using an antennal cDNA library. The full cDNA of HoblOBP3 and HoblOBP4 was cloned using reverse transcription PCR and rapid amplification of the cDNA ends. Homology modeling of both OBPs was performed using SWISS-MODEL on-line tools. Next, the two OBPs were expressed in *Escherichia coli* and purified using Ni ion affinity chromatography. The ligand-binding properties of HoblOBP3 and HoblOBP4 in 42 ligands respectively were measured using the fluorescence probe N-phenyl-naphthylamine (1-NPN). The results obtained from competitive binding assays demonstrated that HoblOBP4 showed a broader range of binding affinities to the test compounds, while HoblOBP3 displays more specific binding affinity. Furthermore, other OBPs and CSPs were expressed in *Escherichia coli* and purified using Ni ion affinity chromatography. Binding curves were measured for binary mixtures of OBPs and CSPs using 1-NPN, and the Scatchard plots exhibited “J”-like nonlinear correlation trends in some samples. In addition, competitive binding assays of the HoblOBP1 and HoblOBP2 mixtures and of the HoblOBP2 and HoblOBP4 mixtures with representative compounds unexpectedly demonstrated good affinity, which revealed extreme differences that were only obtained using the individual proteins. In the immunocytochemical analysis, colocalization of HoblOBP1 and HoblOBP2, and of HoblOBP2 and HoblOBP4, was detected in the sensilla basiconica and sensilla placodea, respectively. All of these results suggested that HoblOBP1 and HoblOBP2, as well as HoblOBP2 and HoblOBP4, may serve as heterodimers in the sensillum lymph.

## Introduction

The sophisticated insect olfactory system can detect and discriminate between different amounts of odorants, which are volatile small organic molecules in the environment. This characteristic property plays a crucial role in insect behaviors, such as host seeking, mating, ovipositing, as well as escape behaviors [1-5]. Indeed, the process of olfactory recognition involves several types of proteins, including odorant binding proteins (OBPs), olfactory receptors (ORs), odorant-degrading enzymes (ODEs), sensory neuron membrane proteins (SNMPs), and ionotropic receptors (IRs) [6]. OBPs exist at a high concentration (up to 10 mM) in the lymph of the antennal sensilla, which surrounds the dendrites of sensory neurons and functions as a carrier for lipophilic odorant molecules [5,7,8]. OBPs are commonly small molecule, water-soluble polypeptides, and exhibit six conserved cysteines that paired with three disulfide bridges in an interlocking fashion [6,9-14]. The first identified insect OBP was found in the giant moth *Antheraea polyphemus* [15]. Thus far, OBPs from more than 40 insect species belonging to eight different orders have been isolated and cloned [7]. However, these OBPs appear to be very divergent from those of other insect orders and are expressed in sensory organs, particularly in the antennae [16,17]. More recently, chemosensory proteins (CSPs), which are members of a second family of soluble polypeptides in insects, have been identified in the lymphs of various chemoreception organs [18-23], as well as in non-chemoreception organs [24-27]. In contrast to OBPs, CSPs are better conserved and more widely distributed in insect species (10 orders) [7,12,22]. Within the last two decades, both classes of soluble proteins have been studied to understand their functions in insect chemoreception [12-14,22].

Although the molecular mechanism of these proteins as filters in the recognition of target odors is not yet completely understood, an olfactory model has been proposed. Two decades worth of reported studies have shown that various lipophilic odorants from the external surroundings can be captured and transported by OBPs into the sensillar lymph to activate ORs to initiate signal transduction [3,4,7]. Until recently, the involvement of OBPs in the recognition of olfactory stimuli has not been completely elucidated [6,7]. There are two exclusive functional patterns of OBPs. The first pattern suggests that ORs can be activated by the odorant itself (which has been observed in moths and in mosquitoes) [7,8]. If this assumption is true, then OBPs might exhibit binding and releasing functions [28-30]. A classical study performed on *Bombyx. mori* PBP showed that conformational changes enabled pheromones to enter the binding pocket in a neutral environment. However, as the pH changes from neutral to acidic, OBP-odorant complexes become unstable, and the pheromone molecule is released from the binding cavity [31,32]. Several similar studies have been performed in other insect orders, such as the OBPs of the cockroach *Leucophaea maderae* [33], giant moth *A. polyphemus*[34], *Amyelois. transitella* [35,36], *Anopholes gambiae* [37], *Aedes. aegypti* [38] and *Culex pipiens quinquefasciatus* [39]. Another pattern has indicated that ORs may be activated by an OBP-odorant complex, and that OBPs might be required for the interaction with ORs in insects [40], about which several experimental evidence has been provided [29,41]. LUSH, an OBP76a in *Drosophila Melanogaster*, is expressed in the sensillum lymph. T1 trichoid sensilla in wild-type flies respond to the aggregation pheromone vaccenyl acetate (VA). However, they cannot detect VA in the absence of the LUSH gene. Further studies have shown that the neuronal sensitivity to VA in the mutant flies may be rescued if LUSH is added in T1 sensilla [42]. This study provides evidence for the requirement of OBPs in olfactory recognition. Although related studies on the two patterns have been reported, this olfactory receptive mechanism is still not well understood. 

A presumed function of the OBP has been previously proposed and indicates that OBPs can form dimers to carry ligands in union [37,43,44]. Although scattered evidence for OBP dimer formation in some insect species has been reported, there is still insufficient evidence for this hypothesis [45-49]. In an early study of OBPs in *A. gambiae*, a three-dimensional structure study has revealed that OBPs are present as dimers, and that their ligand-binding pockets connect from one end of the protein to the other, resulting in a continuous, long hydrophobic tunnel that may potentially allow passage of a ligand [37]. Indeed, this hypothesis is only an extrapolation based on an analysis of the crystal structure and the exceptionally high concentration of OBPs in the sensillar lymph. Additionally, another study on specific interactions among odorant-binding proteins in *A. gambiae* has demonstrated that OBPs are capable of forming homodimers and heterodimers [43]. This result may provide evidence to support the theory of a long hydrophobic tunnel mechanism. Furthermore, one recent study demonstrated unexpected binding characteristics of OBP mixtures (OBP1 and OBP4) in *A. gambiae* using fluorescence binding assays, which revealed OBP heterodimer formation [44]. Moreover, a co-expression study performed in the antennal sensilla of *A. gambiae* was consistent with previous studies. Although evidence of dimer formation in OBPs has been found in *A. gambiae*, whether this interacting mechanism exists *in vivo* or in other species requires further exploration.

Underground pests are a harmful class group in agriculture, and their ability to conceal themselves, and extensive feeding habits result in difficulties in the prevention and control of pests [50-52]. The scarab beetle, *Holotrichia oblita* Faldermann (Coleoptera: Scarabaeidae), belongs to such a class and has caused serious economic damage to crops, fruit trees and forest trees in China [13]. An environmentally friendly method for the control of *H. oblita* is needed. A better understanding of the olfactory processes may help to improve current insect control strategies, particularly those strategies that rely on deviation from their normal behaviors, such as pheromone-based traps [3,4,53,54]. Thus, studies on OBPs in *H. oblita* have quickly developed, and the ability to sequence the genome of the red flour beetle *Tribolium castaneum* has dramatically accelerated these studies [13,52,55]. Some evidence has indicated that OBPs in *H. oblita* and *Holotrichia parallela* are directly involved in the selective perception of volatilizing odors from the host plant and putative sex pheromones [13,52]. In addition, these results demonstrate that OBPs can distinguish between odorants according to their chain length, functional group and alkene geometry [56].

In the present study, we identified two OBPs from the antennae cDNA library of *H. oblita*, HoblOBP3 and HoblOBP4. We expressed both of these OBPs in a heterologous system and measured their ligand-binding activities using a fluorescence competitive binding assay with the N-phenyl-1-naphthyl-amine (1-NPN) fluorescent probe. In addition, other known HoblOBPs and HoblCSPs have been expressed using the same method. Binding curves with binary mixture groups indicated that HoblOBP2 interacted with either HoblOBP4 or HoblOBP1. We detected the co-expression of HoblOBP2 and HoblOBP4 and of HoblOBP2 and HoblOBP1 in the antennal sensilla of *H. Oblita* using double immunolabelling procedures and immunoelectron microscopy. Taken together, our results provide new evidence for olfactory recognition in underground pests.

## Results

### 1 Sequences and homology analysis

**Figure 1:**
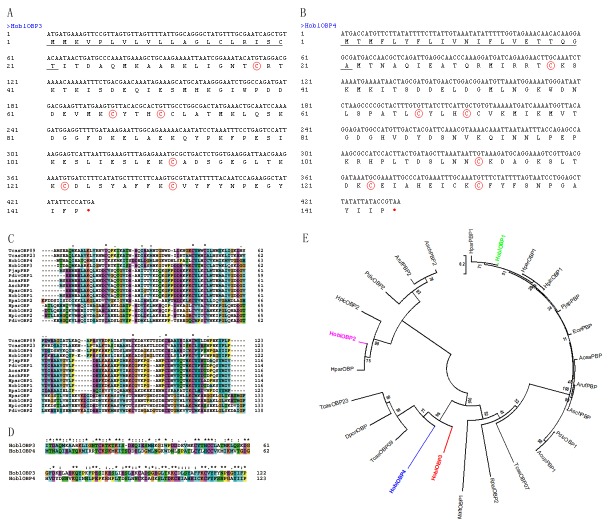
Characterization and phylogenetic analysis of HoblOBP3 and HoblOBP4. (**A**-**B**) The ORF of nucleotide sequence and deduced amino acid sequence of the OBP3 (**A**) and OBP4 (**B**) from H. *oblita*. The six conserved cysteines are indicated in rings with red color. The predicted signal peptide is underlined. The asterisk with red color marks the translation-termination codon. (**C**) Alignment of some OBPs amino acid sequence from Coleoptera insects. (**D**) Alignment of amino acid sequence between HoblOBP3 and HoblOBP4. (**E**) Phylogenetic tree of OBPs amino acid sequences in Coleoptera, including HoblOBPs. The corresponding OBPs in alignment and phylogenetic tree are listed as follow. RpalOBP2 (AAD31875, *Rhynchophorus palmarum*), DponOBP (AFI45058, *Dendroctonus ponderosae*), TcasOBP09 (EFA10713, *Tribolium castaneum*), TcasOBP23 (EFA10803, *Tribolium castaneum*), TcasOBP07 (EFA04593, *Tribolium castaneum*), HparOBP (AEA76516, *Holotrichia parallela*), HparPBP1 (ADF87391, *Holotrichia parallela*), HparOBP1 (BAC07272, *Holotrichia parallela*), HpicOBP1(BAC07270, *Heptophylla picea*), HpicOBP2 (BAC07271, *Heptophylla picea*), PjapPBP (AAC63436, *Popillia japonica*), EoriPBP (BAB70711, *Exomala orientalis*), AosaPBP (AAC63437, *Anomala osakana*), PdivOBP1 (BAA88061, *Phyllopertha diversa*), PdivOBP2(BAA88062, *Phyllopertha diversa*), AcupPBP1 (BAC06496, *Anomala cuprea*), ArufPBP (BAF79995, *Anomala rufocuprea*), ArufPBP2 (BAF91329, *Anomala rufocuprea*), AschPBP(BAF79599, *Anomala schonfeldti*), AschPBP2 (BAF79600, *Anomala schonfeldti*), MaltOBP1 (ABR53888, *Monochamus alternatus*), HoblOBP1(ACX32050, *Holotrichia oblita*), HoblOBP2(ACX32049, *Holotrichia oblita*), HoblOBP3 (ADX96030, *Holotrichia oblita*), HoblOBP4 (ADX96031, *Holotrichia oblita*).

Two partial sequences were obtained from the cDNA library of the *H. oblita* antennae screening. Full-length cDNAs encoding HoblOBP3 and HoblOBP4 were cloned from *H. oblita* and called HoblOBP3 and HoblOBP4 (GenBank IDs: ADX96030 and ADX96031). The ORF of the HoblOBP3 cDNA consisted of 429 nucleotides and encoded 143 amino acids. The predicted signal peptide contained the initial 21 amino acids, as identified using SignalP 4.1 software. The ORF was terminated by a TGA stop codon. The predicted molecular weight and isoelectric point of the mature HoblOBP3 were 16.2 kDa and 6.71, respectively (Figure 1A). The ORF of the HoblOBP4 cDNA consisted of 432 nucleotides and encoded 144 amino acids. The predicted signal peptide contained the initial 21 amino acids. The ORF was terminated by a TAA stop codon. The predicted molecular weight and isoelectric point of the mature HoblOBP4 were 16.3 kDa and 6.87, respectively (Figure 1B). HoblOBP3 and HoblOBP4 contained a typical framework of OBPs (six conserved cysteines paired in three disulfide bridges), which belonged to the classical group of OBPs (Figure 1C-D). The conserved patterns are listed as follows: HoblOBP3: X16-Cys-X27-Cys-X3-Cys-X40-Cys-X10-Cys-X8-Cys-X12, HoblOBP4: X16-Cys-X28-Cys-X3-Cys-X40-Cys-X10-Cys-X8-Cys-X12, of which X represents any amino acid. The frameworks of HoblOBP3 and HoblOBP4 shared a high identity and were consistent with a “signature” for insect OBPs [7]. 

The sequence alignment between HoblOBP3 and HoblOBP4 was performed. These result showed that the amino acid identity received a low score and only reached 38% identity (Figure 1D). Multiple sequence alignment among the HoblOBPs and corresponding OBPs from other species of Coleoptera are shown in Figure 1C. HoblOBP3 and HoblOBP4 shared low identity (

< 41%) with other Coleoptera OBPs. The highest identities of HoblOBP3 and HoblOBP4 were 41% and 38% with TcasOBP09 (*T*. *castaneum*) and 41% and 34% with TcasOBP23 (*T*. *castaneum*), respectively (Figure 1C). These phylogenetic relationships indicated that HoblOBP3 and HoblOBP4 belonged to different branches than HoblOBP1 and HoblOBP2, which were consistent with their sequence alignments (Figure 1E). These results demonstrated the diversity of the HoblOBP family.

### 2 Protein structural analysis

**Figure 2:**
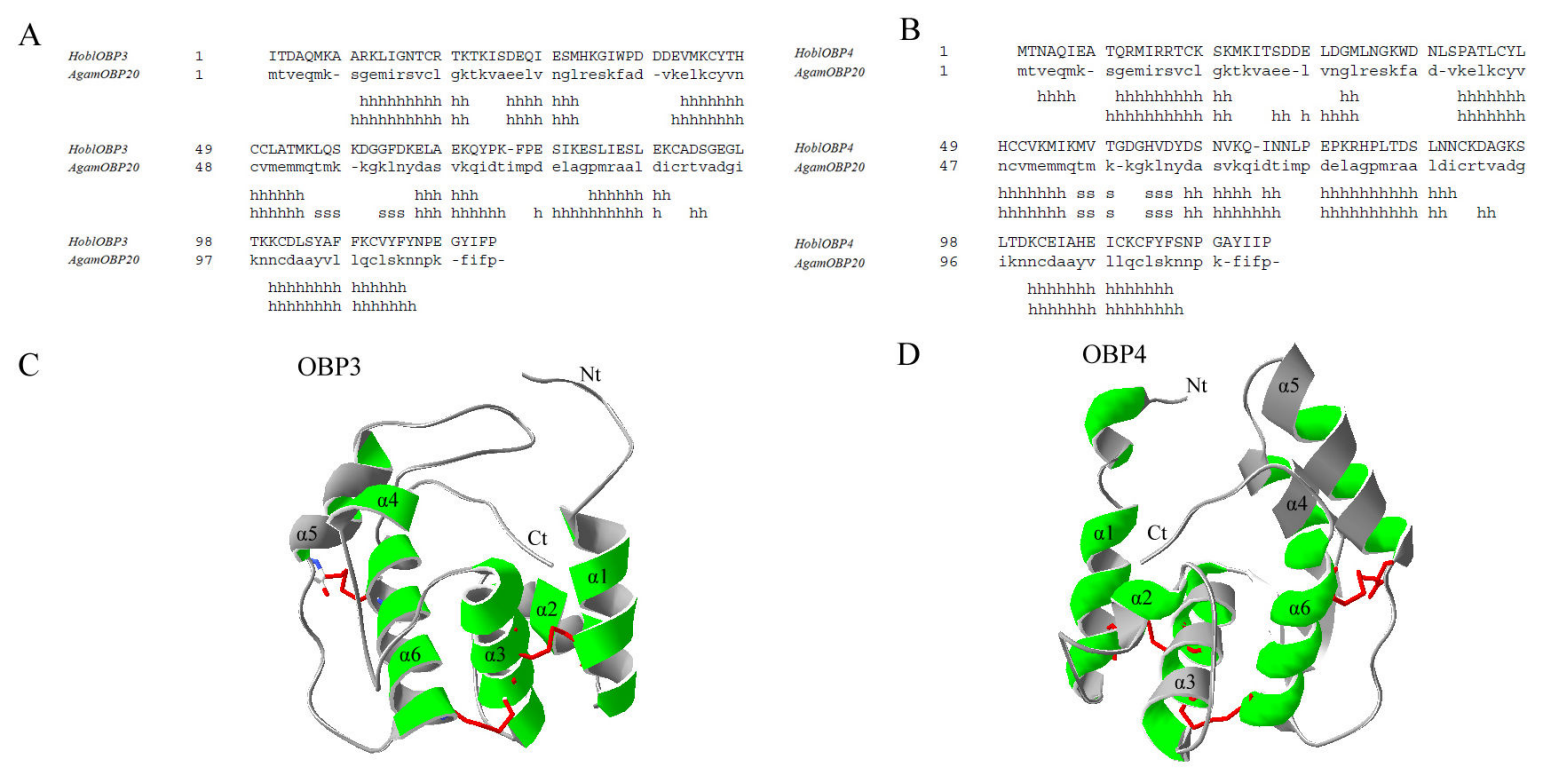
Predicted three-dimensional model of HoblOBP3 and HoblOBP4. Two models both used the crystal structure of *A. gambiae* OBP20 (AgamOBP20) (PDB: 3VB1_A) as a template. (**A**–**B**): Alignments between HoblOBP3 and AgamOBP20 (**A**) or HoblOBP4 and AgamOBP20 (**B**) used in the homologous modeling. The secondary structure elements are shown in the sequence alignments. A small letter “h” in the alignment below the amino acid residues represents the potential α-helix formation. A small letter “s” in the alignment represents the β-sheet formation. (**C**-**D**): the three-dimensional structure of HoblOBP3 (**C**) and HoblOBP4 (**D**) are colored green. The three disulfide bridges are colored red. The N- termini and C- termini as well as α-helices are labeled.

The BLAST analysis was performed against the PDB database to identify suitable templates for the generation of the three-dimensional structures of HoblOBP3 and HoblOBP4. The crystal structure of *A. gambiae* OBP20 (AgamOBP20) (PDB: 3VB1_A) was chosen as a template for both HoblOBP3 and HoblOBP4 [57] The sequence identities between HoblOBP3 and AgamOBP20 and between HoblOBP4 and AgamOBP20 were 26.0% and 26.6%, respectively (Figure 2A-B). The three-dimensional models of HoblOBP3 and HoblOBP4 were predicted using the SWISS-MODEL online tools (Figure 2C-D) [58]. The rationale underlying the model evaluation was based on Ramachandran plot. We found that 82.6% and 14.7% of the HoblOBP3 residues were in the most favored regions and in additional allowed regions, respectively. Moreover, 85.5% and 11.8% of the HoblOBP4 residues were in the most favored regions and in additional allowed regions, respectively. 

The predicted three-dimensional structure of HoblOBP3 and HoblOBP4 consisted of six α-helices and three disulfide bridges, which were paired by six conserved cysteines in an interlocking fashion. In the HoblOBP3 pattern, Cys18-Cys50 connected α1-α3, Cys46-Cys102 connected α3-α6 and Cys91-Cys111 connected α5-α6, while for HoblOBP4, Cys18-Cys51 connected α1-α3, Cys47-Cys103 connected α3-α6 and Cys92-Cys112 connected α5-α6 (Figure 2/C-D). The three-dimensional model of HoblOBP3 and HoblOBP4 presented a large binding pocket, and the C-termini extended into the binding pocket, which included the hydrophobic and hydrophilic residues. 

### 3 Expression and purification of the recombinant proteins

**Figure 3:**
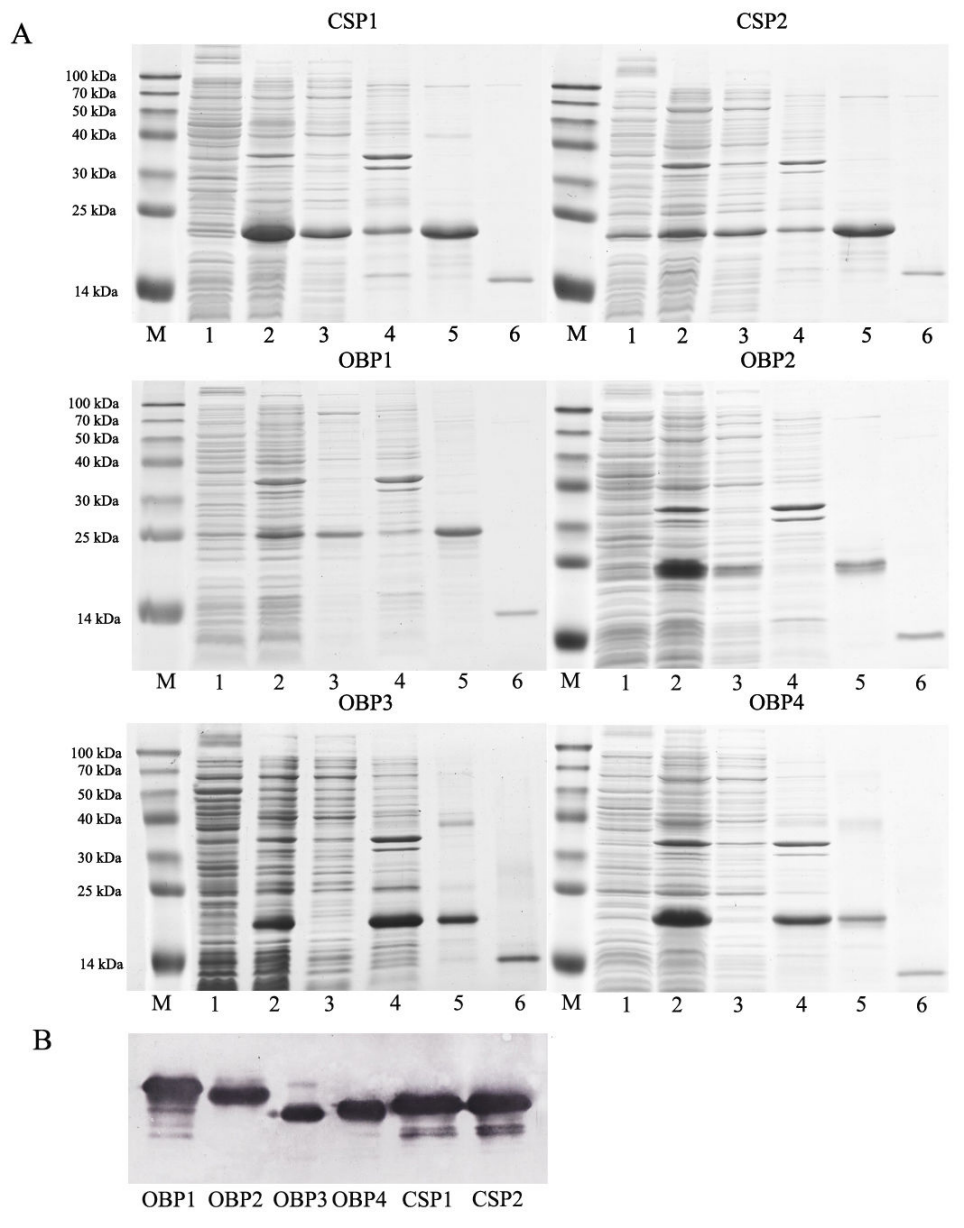
Expression and purification of four OBPs and two CSPs of *H*. *oblita*. SDS-PAGE electrophoretic (15% separation gel) (A) and western blotting (B) analysis of expressed recombine proteins. M: Molecular weight marker of 100, 70, 50, 40, 30, 25, 14 kDa; 1-2: before and after induction of the bacterial culture with IPTG; 3: supernatant; 4: inclusion bodies; 5: Purified fusion protein; 6: Purified protein cleaved His-tag by rEK.

Six recombinant *H. oblita* proteins, including HoblOBP1, HoblOBP2, HoblOBP3, HoblOBP4, HoblCSP1 and HoblCSP2, were expressed in *E. coli* at high yields (more than 20 mg/L). Our recombinant HoblOBP3 and HoblOBP4 proteins were expressed in *E. coli* as inclusion bodies and were solubilized under denatured and reducing conditions, while the other four recombinant proteins were presented in soluble forms. The proteins were then purified using Ni ion affinity chromatography and anion exchange chromatography. The histidine-tag of the recombinant proteins was removed by rEK. SDS-PAGE and Western Blotting were then performed (Figure 3). The purified recombinant proteins were then tested for their binding properties and used in the production of polyclonal antibodies.

### 4 Fluorescence binding assays

**Figure 4:**
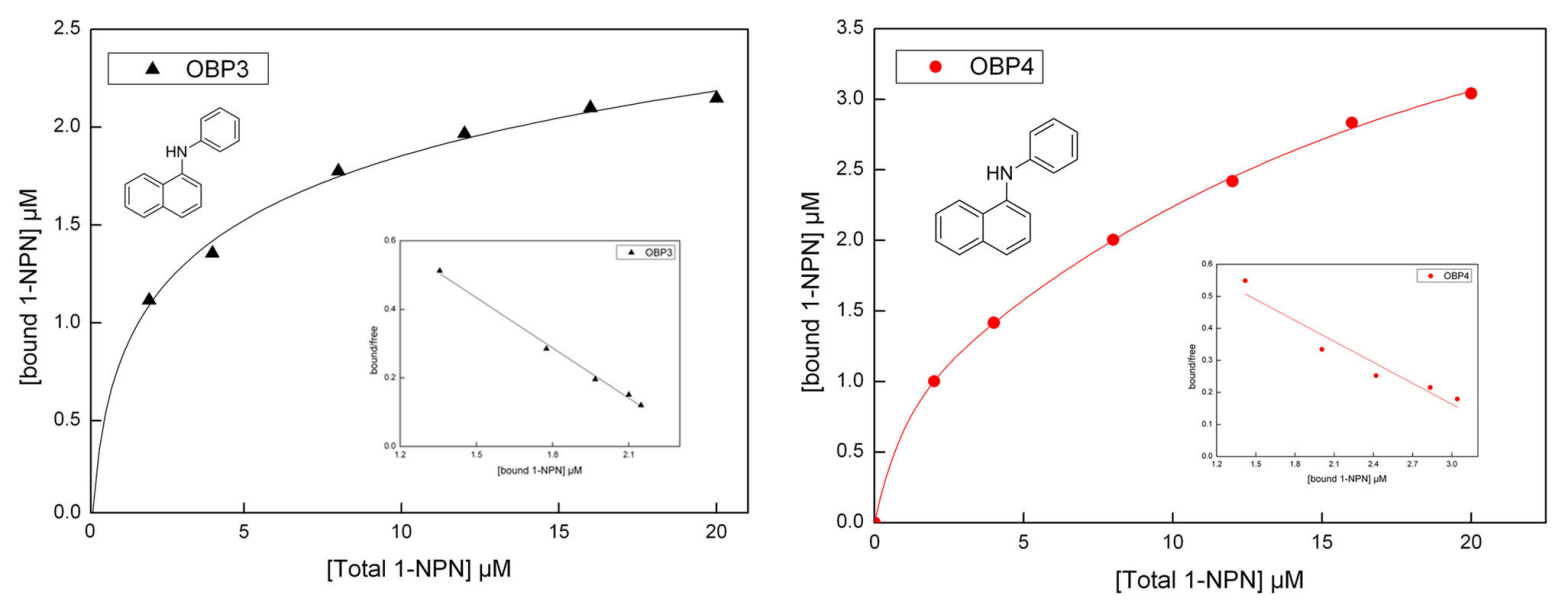
Binding curves of 1-NPN and the relative Scatchard plot. A solution of the protein with 2 µM in Tris-buffer was titrated with 1 mM solution of 1-NPN in methanol, with final concentrations of 2–20 µM. Dissociation constants were HoblOBP3: 1.88 µM; HoblOBP4: 2.78 µM.

Both of the recombinant OBPs (HoblOBP3 and HoblOBP4) was investigated to measure their affinities to a number of potential ligands. 1-NPN was selected as a fluorescent probe to carry out the fluorescent binding experiments [5,13,14]. The dissociation constants were calculated for HoblOBP3 and HoblOBP4, 1.88 µM and 2.78 µM. In both OBPs, a linear profile was obtained from the Scatchard plot (Figure 4).

**Figure 5:**
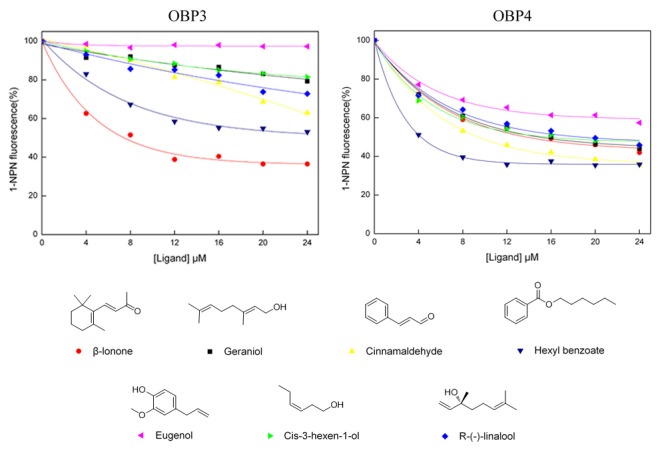
Competitive binding curves of representative ligands to recombinant HoblOBP3 and HoblOBP4. The chemical structures of the ligands are shown below. A mixture of the protein and 1-NPN with both concentration of 2 µM in Tris-buffer was titrated with 1 mM solution of each competing ligand to final concentrations of 4–24 µM.

We selected 42 potential organic compounds on the basis of competitive binding assays, which included compounds from volatile green plants, plant odors, attractant compounds of the scarab beetle species and putative sex pheromones of some beetle species (Table 1) [59-61].In particular, these organic compounds were identified on the basis of differences in chain length, functional group and alkene geometry [56]. The IC50 values (the concentration of ligand at half of the initial fluorescence value), the inhibition constants *K*_*i*_ (for each OBP/ligand combination) and the fluorescence intensity (*Int*) (at the ligand concentration (24 µM) represented by the percentage of the initial fluorescence in the absence of a competitor) are summarized in Table 1. Binding curves of a few representative competition experiments (including plant volatiles and putative sex pheromone) are shown in Figure 5.

**Table 1:** Binding of pure organic compounds to selected recombinant OBPs of *H. oblita*.

**Ligands**	**HoblOBP3**			**HoblOBP4**		
	**IC50**	***Int***	***K_i_***	**IC50**	***Int***	***K_i_***
**Aliphatic alcohols**						
1-Hexanol	-	91	-	12	45	8.8
*trans*-2-Hexenol	-	82	-	24	51	17.7
*cis*-2-Hexen-1-ol	-	70	-	14	42	10.3
*cis*-3-Hexen-1-ol	-	81	-	17	46	12.5
1-Heptanol	-	70	-	21	46	15.4
1-Octen-3-ol	-	86	-	40	54	29.4
1-Nonanol	-	71	-	16	46	11.8
4-tert-Butyl cyclohexanol	-	68	-	19	49	14.0
Retinol	-	-	-	-	-	-
**Aliphatic aldehydes**						
1-Pentanal	41	65	26.8	16	44	11.8
1-Hexanal	-	68	-	15	42	11.0
trans-2-Hexenal	-	74	-	11	39	8.1
1-Heptanal	-	65	-	-	64	-
1-Decanal	-	86	-	-	68	-
**Aliphatic ketones**						
2-Cyclohexen-1-one	-	70	-	23	49	16.9
6-Methyl-5-hepten-2-one	-	78	-	32	52	23.5
**Aliphatic alkanes**						
Heptane	-	63	-	16	43	11.8
Octane	-	70	-	25	51	18.4
Nonane	-	-	-	-	-	-
Decane	-	-	-	-	-	-
**Aliphatic ester**						
Glycine ethyl ester	-	76	-	24	50	17.6
L-Isoleucine methyl ester	-	68	-	24	49	17.7
L-Proline ethyl ester	-	70	-	20	47	14.7
(Z)-3-Hexenyl acetate	-	78	-	40	56	29.4
Propyl benzoate	-	81	-	20	48	14.7
Butyl Benzoate	-	69	-	11	40	8.1
Hexyl benzoate	-	53	-	4	36	2.9
**Terpenoids**						
Geraniol	-	79	-	16	44	11.8
R-(-)-Linalool	-	73	-	20	46	14.7
Myrcene	-	74	-	-	56	-
Terpinen-4-ol	-	75	-	21	48	15.4
α-Terpineol	-	71	-	35.5	53	26.1
Limonene	-	56	-	-	52	-
α-Ionone	16	41	10.4	19	45	14.0
β-Ionone	8	36	5.2	15	42	11.0
β-Caryophyllene	-	-	-	-	-	-
**Aromatic compounds**						
Benzaldehyde	-	68	-	17	44	12.5
Phenethyl alcohol	-	66	-	28	52	20.6
Menthyl salicylate	-	75	-	-	54	-
Benzeneacetaldehyde	-	-	-	-	-	-
Cinnamaldehyde	46	64	30.0	9	36	6.6
Eugenol	-	97	-	-	57	-

Solution of protein and 1-NPN, both at concentration of 2 µM, was in line with the dissociation constants of HoblOBPs/1-NPN complex calculated. Then the mixed solution was titrated with 1 mM solution of each ligand in methanol to final concentrations of 2–50 µM. For each protein, we report the fluorescence intensity (*Int*) measured at the ligand concentration (24 µM) as percent of the initial fluorescence, the concentration of ligand halving the initial fluorescence intensity (IC50), where applicable, and the relative dissociation constant (*K*_*i*_) calculated as described in ‘‘Materials and methods’’. Dissociation constants of ligands whose IC50 exceeded 50 µM are represented as ‘‘-’’.

Similar to related studies performed with other OBPs in the scarab beetle, these two proteins showed clear preferential binding specificities to the ligands examined. While HoblOBP3 appeared to strongly bind to only a few ligands, HoblOBP4 exhibited a broader spectrum of activity and well bound aliphatic and aromatic compounds consisting of 4–13 carbon atoms. The compounds, 1-hexanol, trans-2-hexenal, butyl benzoate, hexyl benzoate and cinnamaldehyde, showed high binding affinities to HoblOBP4 with *K*_*i*_ values of 8.8, 8.1, 8.1, 2.9 and 6.6 µM，respectively, while only α-ionone and β-ionone displayed binding affinities to HoblOBP3 with *K*_*i*_ values of 10.4 and 5.2 µM, respectively. A large number of aliphatic compounds were tested in competition experiments, which revealed moderate binding affinity (Table 1).

**Figure 6:**
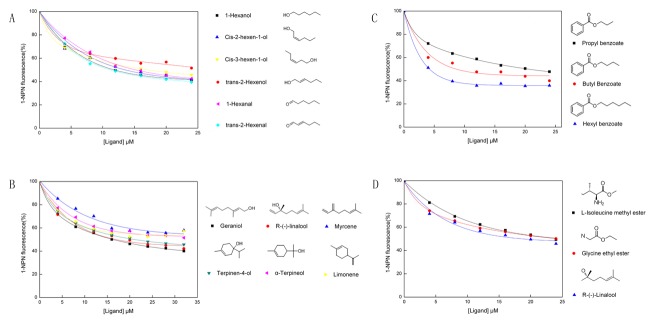
Competitive binding curves of characteristic ligands to recombinant HoblOBP4. The chemical structures of the ligands are shown on the right. (**A**) Competitive binding curves of aliphatic alcohols and aldehydes with six carbon numbers. (**B**) Competitive binding curves of different structural on terpenoids with ten carbon numbers. (**C**) Competitive binding curves of aliphatic ester with different carbon numbers. (**D**) Competitive binding curves of the sex pheromone component.

Interestingly, good affinities for HoblOBP4 were observed for aliphatic alcohols and aldehydes with carbon numbers of six, particularly those containing an insertion of two double bonds into trans-2-hexenal, which restored high binding activity (Figure 6A). For terpenoids with carbon numbers of ten and an open-chain molecular structure, its affinity was higher than for terpenoids with a ring-shaped structure (Figure 6B). In addition, good affinity for HoblOBP4 was also found in open-chain structure compounds bearing other functional groups, such as a hydroxyl group (Figure 6B). Another remarkable observation involved the enhanced affinity demonstrated by aliphatic ester groups compared to other aliphatic groups, which showed a drastically increased affinity with as the carbon number increased; these groups included propyl benzoate, butyl benzoate and hexyl benzoate (Figure 6C). Putative sex pheromone compounds of some beetle species, such as L-isoleucine methyl ester, R-(-)-linalool and glycine ethyl ester, may bind to HoblOBP4 (Figure 6D).

### 5 Fluorescence binding assays with binary protein mixtures

**Figure 7:**
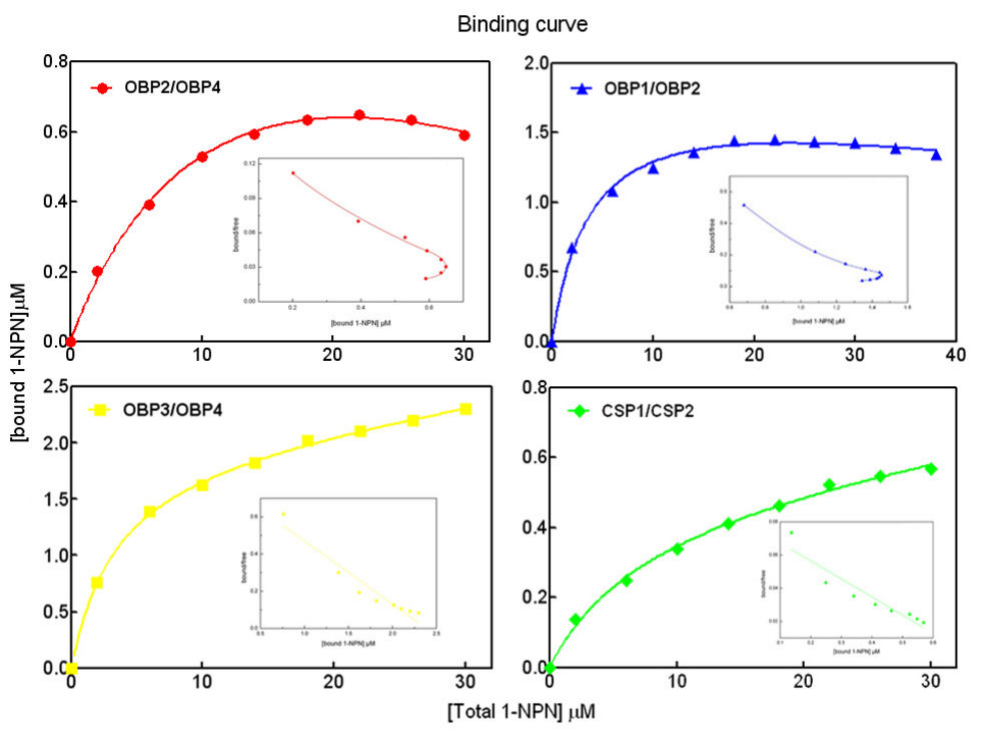
Binding curves of 1-NPN and the relative Scatchard plot using binary protein mixtures. A solution of the protein mixtures (1:1) with 2 µM in Tris-buffer was titrated with 1 mM solution of 1-NPN in methanol, with final concentrations of 2–30 µM.

It is well known that the concentration of OBPs (also likely CSPs) in the sensillum lymph of the insect is extremely high (reportedly 10 mM) [7]. A hypothesis has been proposed that OBPs (or CSPs) homodimers or heterodimers might form [7,43,44]. Thus, all binary potential intersections with four HoblOBPs and two HoblCSPs were tested in competitive binding assays to determine the existence of dimers. These experiments assumed that the protein was 100% active, a stoichiometric ratio between the protein and ligand was 1:1 at saturation and the two proteins were present in equimolar amounts. The representative binding results of 1-NPN to OBP binary mixtures are shown in Figure 7. The binding curves and Scatchard plots of OBP3 and OBP4 mixture or CSP1 and CSP2 mixture were consistent with the functions of the individual proteins. However, the binding curve of the OBP2 and OBP4 mixture or OBP1 and OBP2 mixture presented a different tendency from those obtained with the individual proteins. Moreover, the Scatchard plot exhibited a “J”-like nonlinear correlation trend. Thus, we speculated that the decreased binding velocity was the main cause underlying this phenomenon. When one of the binary protein mixtures demonstrated a good affinity, it would first bind to the fluorescent probe 1-NPN and initially display a rapid upstroke on the binding curve diagram. In contrast, the other proteins from the mixture that exhibited weak binding affinity to 1-NPN and a delayed binding velocity, showed a downward trend at higher concentrations on the binding curve diagram. Such binding of the OBP2 and OBP4 mixture or OBP1 and OBP2 mixture in *H. oblita* was consistent with the OBP1 and OBP4 mixture in *A. gambiae* [44]. In particular, some experiments also indicated interactions between OBP1 and OBP4 in *A. gambiae* [43]. Thus, the hypothesis on a potential interaction between OBP2 and OBP4 (or OBP1 and OBP2) in *H. oblita* was proposed.

**Figure 8:**
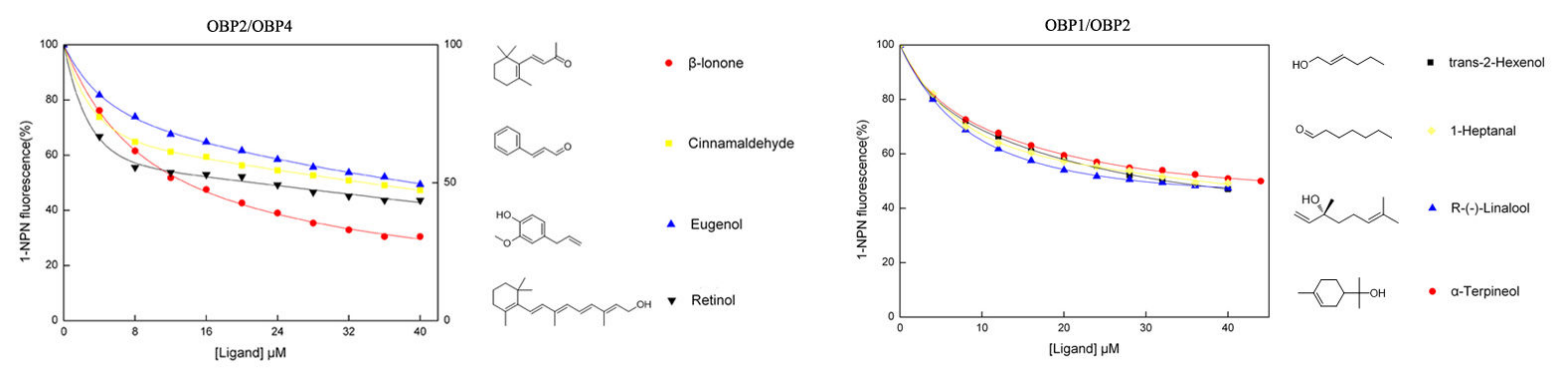
Competitive binding curves of representative ligands to binary protein mixtures OBP2-OBP4 and OBP1-OBP2 in *H*. *oblita*. The chemical structures of the ligands are shown on the right. A mixture of the proteins (equimolar amounts) and 1-NPN with both concentration of 2 µM in Tris-buffer was titrated with 1 mM solution of each competing ligand to final concentrations of 4–40 µM.

We selected four representative organic compounds, including β-ionone, cinnamaldehyde, eugenol and retinol, on the basis of the results obtained from the competitive binding assays with OBP2 and OBP4 mixtures in *H.oblita* (Figure 8). Cinnamaldehyde, eugenol and retinol have been previously reported to demonstrate low-afﬁnity or non-afﬁnity, and β-ionone has been shown to exhibit good afﬁnity for HoblOBP2 [13]. In contrast, cinnamaldehyde and β-ionone showed good afﬁnity, and the remaining two compounds showed no afﬁnity for HoblOBP4 in our study. When we investigated the HoblOBP2 and HoblOBP4 mixtures, we found the good affinity exhibited on these four compounds. β-ionone displayed an enhanced affinity with OBP2 and OBP4 mixtures compared to the individual protein. Surprisingly, good affinity was measured for retinol with HoblOBP2 and HoblOBP4 mixtures, which extremely differed from the individual protein (neither HoblOBP2 nor HoblOBP4 could bind to retinol alone). This result indicated that retinol could bind in a heterodimeric manner with OBP2/OBP4 in *H. oblita*.

A similar assay was performed in HoblOBP1 and HoblOBP2 mixtures with trans-2-hexenol, 1-heptanal, R-(-)-linalool and α-terpineol (Figure 8). Competitive binding assays of HoblOBP1 or HoblOBP2 alone with these four compounds have been previously tested, which showed low-afﬁnity or non-afﬁnity, respectively [13]. Interestingly, an enhanced affinity on HoblOBP1 and HoblOBP2 mixtures compared to HoblOBP1 or HoblOBP2 alone was observed in these four compounds, which was consistent with the results of the HoblOBP2 and HoblOBP4 mixtures.

### 6 Colocalization immunocytochemistry

**Figure 9:**
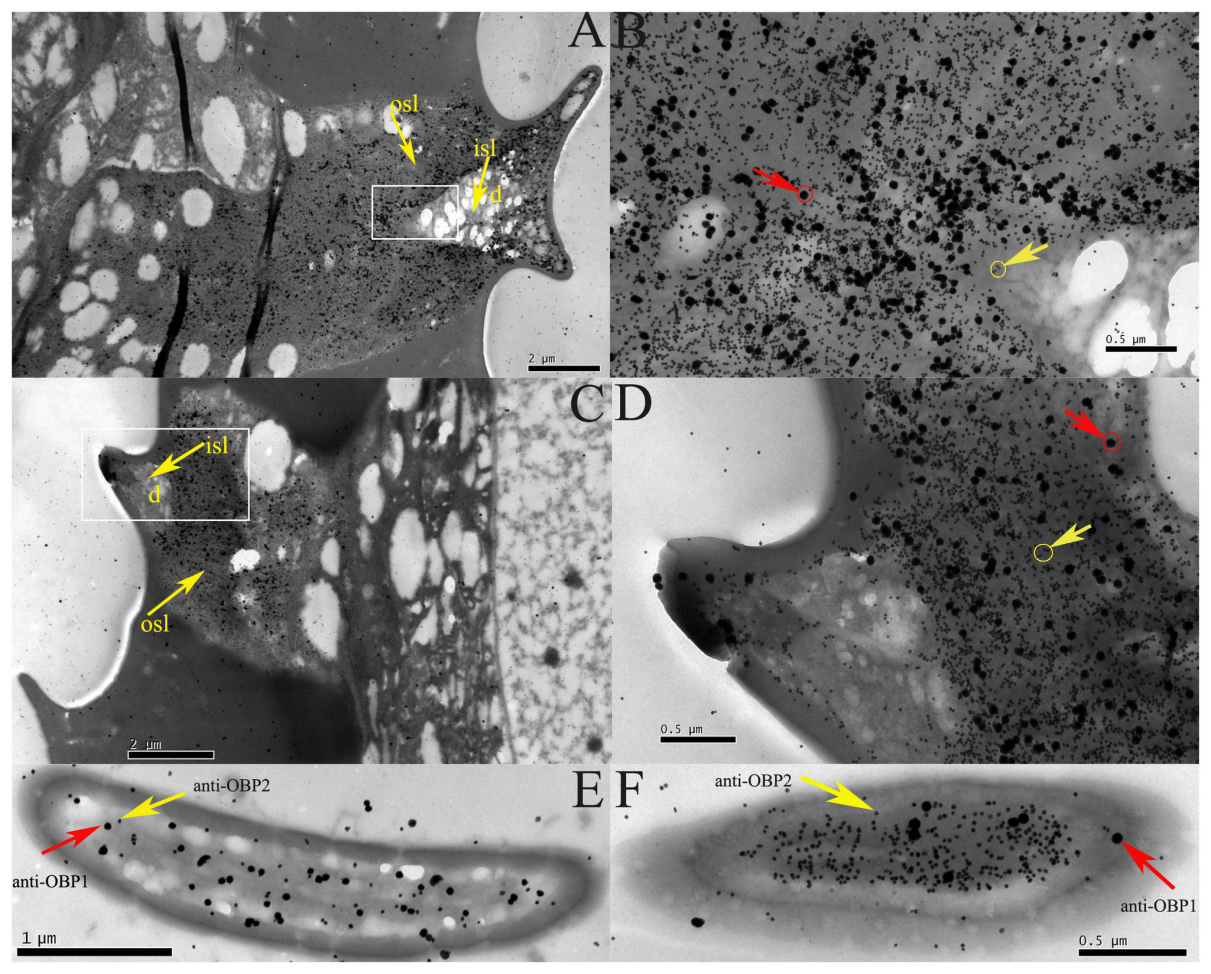
Immunocytochemical colocalization of OBP1-2 in the olfactory sensilla of adult *H*. *oblita*. The coexpressed of HoblOBP1-2 in Sensilla placodeum (A, B, and E) and Sensilla basiconica (C, D, and F) was detected by double labelling of colloidal gold immunocytochemistry. The anti-OBP1 was labeled by the 10-nm gold marker (large silver-intensified granules, with red arrows in B, D, E, and F) and anti-OBP2 was labeled by 5-nm gold marker (small silver-intensified granules, with yellow arrows in B, D, E, and F). B and D was enlargement of part of A and C in white pane respectively. Inner (isl) and outer (osl) sensillum lymph of the dendrites (d) was marked with yellow arrows in A and C.

**Figure 10:**
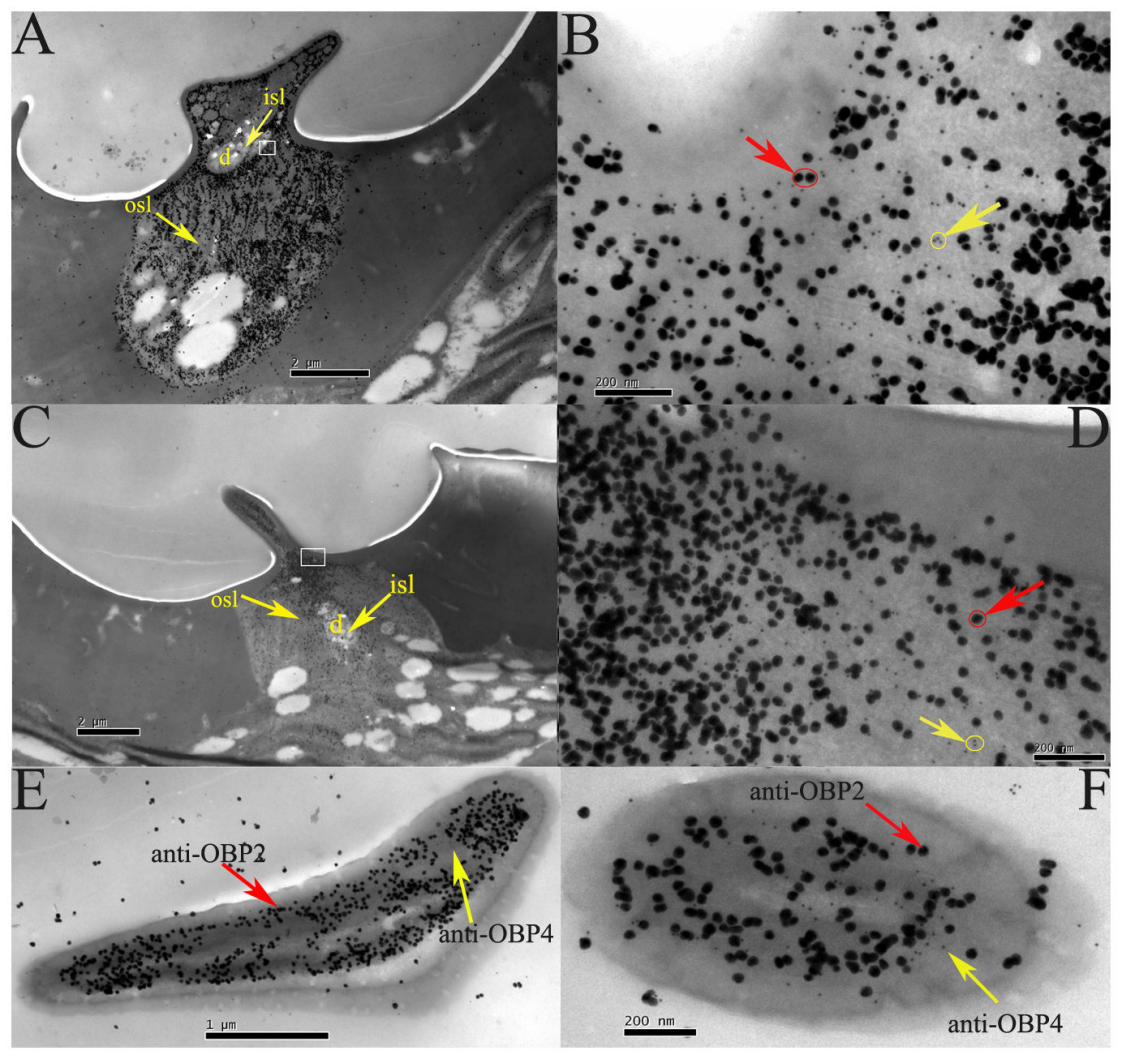
Immunocytochemical colocalization of OBP2-4 in the olfactory sensilla of adult *H*. *oblita*. The coexpressed of HoblOBP2-4 in Sensilla placodeum (A, B, and E) and Sensilla basiconica (C, D, and F) was detected by double labelling of colloidal gold immunocytochemistry. The anti-OBP2 was labeled by the 10-nm gold marker (large silver-intensified granules, with red arrows in B, D, E, and F) and anti-OBP4 was labeled by 5-nm gold marker (small non-intensified granules, with yellow arrows in B, D, E, and F). B and D was enlargement of part of A and C in white pane respectively. Inner (isl) and outer (osl) sensillum lymph of the dendrites (d) was marked with yellow arrows in A and C.

Double-labeling for HoblOBP1 and HoblOBP2 (Figure 9) and for HoblOBP2 and HoblOBP4 (Figure 10) was performed using colloidal gold post-embedding immunocytochemistry. Polyclonal antiserums of HoblOBPs (anti-OBP1, anti-OBP2, and anti-OBP4) were used to determine the cellular localization of HoblOBPs in the adult antennae. For double-labeling of HoblOBP1 and HoblOBP2, anti-OBP1 was labeled using a 10-nm gold marker (large silver-intensified granules), and anti-OBP2 was labeled using a 5-nm gold marker (small silver-intensified granules), which are shown in Figure 9B, D, E, and F. Similarly, the double-labeling of HoblOBP2 and HoblOBP4 are shown in Figure 10B, D, E, and F. Different chemosensory sensilla, sensilla placodea (Figure 9 and 10A, B, and E) and sensilla basiconica (Figure 9 and 10C, D, and F) of both sexes were strongly labeled by the two protein groups (HoblOBP1 and HoblOBP2 or HoblOBP2 and HoblOBP4), suggesting that each protein group was coexpressed in these same sensilla. The outer sensillum lymph (osl) surrounding the dendrites (d) was robustly labeled (Figure 9 and 10A–D), while the inner sensillum lymph (isl) of the dendrites was never labeled (Figure 9 and 10A, C).

## Discussion

Full-length cDNAs encoding HoblOBP3 and HoblOBP4 were cloned using RACE-PCR. The deduced amino acid sequence suggested that these two proteins consisted of a typical framework of OBPs (six-conserved cysteines) and may be new members of the OBP family in *H. oblita* but share a low sequence similarity from other species of Coleoptera, including HoblOBP1 and HoblOBP2. In fact, Coleoptera is the biggest order in insecta. However, reported OBPs in Coleoptera are very rare. Since the first genome sequences of the red flour beetle *T. castaneum* has been sequenced [55], a growing number of OBPs have been found in Coleoptera [13,52] and more will surely be discovered and their diversified functions revealed in the future.

Our fluorescent binding experiments provided interesting results. In general, HoblOBP4 exhibits a broader affinity compared to HoblOBP3 in response to the ligands assayed. Such binding specificity of HoblOBP3 with α-ionone and β-ionone is remarkable when compared with the broad spectrum of binding to other insect OBPs, as previously reported in the literature [12-14], While HoblOBP4 demonstrated a broader spectrum of activity, it exhibited good binding with aliphatic and aromatic compounds containing 4–13 carbon atoms, particularly with hexyl benzoate. Interestingly, we observed an enhanced affinity of HoblOBP4 for aliphatic esters compared to other aliphatic groups; its affinity increased drastically as the carbon number increased (i.e., propyl benzoate, butyl benzoate and hexyl benzoate). These results were consistent with previous reports of HoblOBP1 and HoblOBP2 [13]. In our binding assay, β-ionone, a strong ligand, showed a higher affinity compared to α-ionone for both HoblOBP3 and HoblOBP4, which suggests that isomers constitute one factor that influence affinity in fluorescence binding experiments. In another analysis of our experiment, the effect on the length difference of the carbon chains was reflected in the binding affinity. In general, the ligand affinities decreased when the number of carbon atoms increased, which was mainly observed in the aliphatic alkanes. Moreover, good affinity was measured for aliphatic alcohols and aldehydes with carbon numbers of six in HoblOBP4, particularly the insertion of two double bonds in the trans-2-hexenal, which restored high binding activity. For terpenoids with carbon numbers of ten, its affinity for an open-chain molecular structure was higher compared to that for terpenoids with a ring-shaped structure. In addition, good affinity was also observed with open-chain structure compounds bearing other functional groups, such as a hydroxyl group. Thus, the good affinity observed in geraniol can be easily explained. Furthermore, cinnamaldehyde exhibits good affinity, which may be attributed to its functional groups (the presence of double bonds or an aldehyde group). In contrast, β-caryophyllene exhibited a much weaker affinity, which could be attributed to its large ring structure. Thus, the position and variety of functional groups may considerably affect the binding affinity. Briefly, our fluorescent binding experiments were consistent with other previously reported insect OBPs, and chain length, functional group and alkene geometry were the main impact factors that affected binding affinity [5,12-14,56].

In our study, the three-dimensional structure of HoblOBP3 and HoblOBP4 was predicted respectively. The crystal structure of AgamOBP20 was selected as the template for both HoblOBP3 and HoblOBP4 [57]. It has been shown that its C-terminus folds back into the protein core, which was similar in structure to AgamOBP1 [37]. Such a conformation is similar to the structures of other OBPs, which have been previously published in the honeybee *Apis mellifera* (AmelASP1), mosquito *A. aegypti* (AaegOBP1), and mosquito *C. quinquefasciatus* (CquiOBP1) [38,39,62]. However, these OBPs did not form a seventh α-helix in the C-terminus and have different binding mechanisms in the silkworm *B. mori* PBP [6]. It may be assumed that if similar structures correspond to the same mechanism of binding and releasing, then HoblOBP3 or HoblOBP4 will be consistent with these OBPs. Moreover, we also predicted the three-dimensional structure of HoblOBP1 and HoblOBP2, which were similar to HoblOBP3 and HoblOBP4. The related analysis of ligands binding site with these HoblOBPs will be issued in another paper (Zhuang XJ et al, in preparation). It will further explain the interacted mechanism between ligands and proteins.

An alternative hypothesis that has been proposed involves the protein-binding pocket and its ability to form a long hydrophobic tunnel from one end of the protein to the other (present as a dimer), which may potentially allow ligands to freely pass through the protein channel [37]. Given the exceptionally high concentration (10 mM) reported for OBPs in the sensillar lymph with individual proteins that nearly touching one another, it is likely that the hydrophobic ligand could pass from one OBP to other until it reaches to the ORs [7]. This model, which is suggested by the AgamOBP1 structure, is presented as a dimer [37]. In the same year, another study on specific interactions among odorant-binding proteins of *A. gambiae* has demonstrated that two OBPs, OBP1 and 4, are capable of forming heterodimers [43]. Subsequently, another study also indicated that the unexpected binding characteristics of AgamOBP1 and AgamOBP4 mixtures, as measured using fluorescence binding assays, could be interpreted as the heterodimeric formation of OBPs [44]. All of these studies provide convincing evidence for a hydrophobic tunnel hypothesis. Interestingly, HoblOBP1 and HoblOBP2 are structurally analogous to AgamOBP1 (unpublished data), and our experimental results demonstrate that the binding characteristics of HoblOBP1 and HoblOBP2 mixtures, as measured using fluorescence binding assays, are consistent with the AgamOBP1 and AgamOBP4 mixtures, which exhibit an unusual tendency. The competitive binding assays of HoblOBP1 and HoblOBP2 mixtures were performed with some representative organic compounds. These results show that an enhanced affinity is exhibited in HoblOBP1 and HoblOBP2 mixtures compared to either HoblOBP1 or HoblOBP2 alone. Moreover, parallel colocalization analysis indicated that OBP1 and OBP2 are co-expressed in the same sensilla. Thus, potential dimer formation between HoblOBP1 and HoblOBP2 has been proposed to support the hypothesis of a long hydrophobic tunnel in *H. oblita*. Such a presumption may also be pertinent for the dimer formation between HoblOBP2 and HoblOBP4 and may be another piece of evidence supporting good affinity for retinol in HoblOBP2 and HoblOBP4 mixtures, although retinol demonstrated good affinity with neither HoblOBP2 nor HoblOBP4 alone. It is suggested that retinol might be bound by the heterodimeric OBP2/OBP4 in *H. oblita*. In the general case, the binding affinities of these ligands decreased when the number of carbon atoms increased [13,14]. If so, it is possible that the recognition mechanism of the insect for multi-carbon macromolecular compounds may be realized by dimer transportation. We deduce that HoblOBP2 and HoblOBP4 form heterodimers, while HoblOBP1 formed homodimers in the former study [13]. Similar to the hypothesized hydrophobic protein tunnel in AgamOBP1, it is likely that a long hydrophobic tunnel consisting of OBP heterodimers or homodimers will transport the ligand to the ORs if an identical ligand can be recognized from multiple HoblOBPs.

In this study, coexpression was observed at the subcellular level by immunocytochemical localization of two OBP groups within the *H. oblita* antennae. Importantly, a large number of studies on OBPs using polyclonal antibodies were performed in Lepidoptera. As a general rule, the sensilla trichodea express PBPs, while most sensilla basicona, which respond to general odorants, mainly express GOBPs [63-66]. However, a colocalization study performed in the same sensillum was completed using two OBPs of *Drosophila*: OS-E and OS-F [67]. The resulting study confirmed three Drosophila OBPs (LUSH, OS-E and OS-F), which were coexpressed in the sensilla trichodea, using colloidal gold post-embedding immunocytochemistry [68]. Similar conclusions regarding their colocalization were confirmed in *A. gambiae* OBPs [44]. In a previous study of OBPs in *H. oblita*, consecutive sections labeled with anti-OBP1 and anti-OBP2 antisera, respectively, illustrated that HoblOBP1 and HoblOBP2 were both expressed in the sensilla placodea and basiconica [13]. Colloidal gold granules of different sizes were used to confirm the colocalization of HoblOBP1 and HoblOBP2 in the same sensillum. Such circumstances also apply to HoblOBP2 and HoblOBP4. Importantly, HoblOBP1 and HoblOBP2, as well as HoblOBP2 and HoblOBP4, are co-expressed in sensilla placodea and basiconica, respectively, which are likely to shift toward the functional relevance of heterodimers in the sensillum lymph. It has been suggested that the co-expression of different OBPs within the same sensillum may potentially broaden the range of odorants to which the olfactory receptor neurons can respond [13,69]. Thus, the colocalization between HoblOBPs strongly supports the hydrophobic tunnel hypothesis.

Our binding assay and colocalization studies support that OBPs can effectively perceive plant volatiles or pheromones by forming heterodimers in the sensillum lymph and potentially expanding their chemical communication, which are consistent with hydrophobic tunnel hypothesis. Thus far, several types of mechanisms underlying olfactory recognition have been proposed to explain the insect’s physiological functional and behavioral responses. All of these hypotheses contribute to a better understanding of olfactory processes in insects, which can facilitate the development of strategies directed towards disrupting specific behaviors in pest species.

## Materials and Methods

### 1 Insects and reagents

The scarab beetle *H. oblita* was provided by Cangzhou Academy of Agriculture and Forestry Sciences, Cangzhou city, Hebei province, China. When the scarab beetle breaks out, it was collected in the test field from Cangzhou Academy of Agriculture and Forestry Sciences. This collection of *H. oblita* is permitted by the committee of Biology of Plant Diseases and Insect Pests of Cangzhou Academy of Agriculture and Forestry Sciences. The adult antennae were dissected in 0.75% NaCl saline solution and immediately frozen in liquid nitrogen. The isolated antennae were stored at –70°C until use.

### 2 Screening of OBP genes in the antennal cDNA library

Total antennal RNA was isolated from 100 antennae of *H. oblita* (females) using Trizol reagent (Invitrogen, Carlsbad, CA, USA). The antennal cDNA library was constructed using the Creator^TM^ SMART^TM^ cDNA Library Construction Kit (Clontech, Mountain, CA, USA), according to the manufacturers’ protocol. Single clones were picked and sequenced after being inserted into a vector (TaKaRa Co., Dalian, China). The partial sequences of the OBP genes were identified using BlastX.

### 3 Cloning and sequencing

The overall lengths of the cDNA sequences were obtained by performing rapid-ampliﬁcation of cDNA ends (RACE), according to the instructions of the 5'- Full RACE Kit and 3'-Full RACE Core Set Ver.2.0 (Takara Co., Dalian, China). The 5' and 3' RACE gene-speciﬁc primers (GSPs) were designed from the partial coding sequences of HoblOBP3 and HoblOBP4 and synthesized by TaKaRa Company (Dalian, China). These primer sequences are listed in Table 2. Polymerase chain reaction (PCR) was performed using ExTaq DNA polymerase (Takara Co., Dalian, China) under the same conditions, including a pre-denaturation step (94°C for 3 min), 30 cycles (94°C for 30 s, 55°C for 30 s and 72°C for 1 min) and further extension (72°C for 10 min). The PCR products were digested and ligated into the pGEM-T Easy Vector (Promega, Madison, WI). The recombinant plasmid was transformed into *E. coli* DH5α competent cells and plated onto LB solid medium/ ampicillin. Positive clones were selected for the sequence using the dideoxynucleotide chain termination method (TaKaRa Co., Dalian, China). 

**Table 2:** Oligonucleotide primers used for amplifying OBP3 and OBP4 genes in *H. oblita*.

Protein category	Primer name	Sequence
OBP3	3' RACE GSP1	5' -CCAGATGATGACGAAGTTATGAAG-3'
	3' RACE GSP2	5' -TACACGCACTGTTGCCTGG-3'
	5' RACE GSP1	5' -CCTTCACCAGAGTCAGCGCAT-3'
	5' RACE GSP2	5' -ATTCTTTATCAAAACCTCCATC-3'
	Forward(partial)	5' -ATAACTGATGCCCAAATGAT-3'
	Reverse(partial)	5' -TGGGAATATATAGCCTTCTGGATTG-3'
	Forward	5' - GAATTCATGATGAAAGTTCCGTTAGTG
	Reverse	5' - CTCGAGTCATGGGAATATATAGCCTTCTGGA
OBP4	3' RACE GSP1	5' -CTAAGCCCCGCTACTTTGTGTT-3'
	3' RACE GSP2	5' -CAAAATGGTTACAGGAGATGG-3'
	5' RACE GSP1	5' -CGACTTTCCTGCATCTTTAC-3'
	5' RACE GSP2	5' -TGGCGCTTTGGCTCTGGTAA-3'
	Forward(partial)	5' -ATGACCAACGCTCAGATTG-3'
	Reverse(partial)	5' -GGTATAATATAAGCTCCAGGATTA-3'
	Forward	5' - GAATTCATGACCATGTTCTTATATTTTC
	Reverse	5' - CTCGAGTTACGGTATAATATAAGCTCCAG

GAATTC and CTCGAG presents restriction enzyme cutting site, EcoRI and XhoI, respectively.

### 4 Sequences and structural analysis

When complete the coding sequences of HoblOBP3 and HoblOBP4 obtained using the RACE method, the open reading frames (ORFs) were deduced using the Open Reading Frame Finder (http://www.ncbi.nlm.nih.gov/gorf/gorf.html). The putative signal peptides were predicted using the SignalP 4.1 Server [70]. The molecular weights of the proteins were predicted using SWISS-PROT (http://www.expasy.org/compute_pi). Several OBP sequences of Coleopteran insects were downloaded from the GenBank sequence database and aligned using CLUSTALX 2.0.7[71]. The phylogenetic tree was constructed with MEGA 4.0 (using a neighbor-joining method), and the samples were bootstrapped 1000 times [72].

Three-dimensional models of HoblOBP3 and HoblOBP4 were predicted using the SWISS MODEL on-line tools (http://swissmodel.expasy.org/) [73]. Both HoblOBPs were used on the basis of the structure with the highest alignment score as a template to construct three-dimensional models. Models were manipulated using the Swiss-Pdb Viewer 4.1.0 Server [73]. The rationale underlying the established model evaluation was based on a Ramachandran plot [74].

### 5 Recombinant expression and purification

Gene-specific primers were designed to clone the coding regions of HoblOBP3 and HoblOBP4. The related specific primers with XhoI and EcoRI restriction enzymes site are listed in Table 2. The coding nucleotide sequences were first cloned into the pGEM-T easy vector (Promega, Madison, WI) and digested by XhoI and EcoRI enzymes. The digested products were then ligated into the pET30a (+) expression vector (Novagen, Madison, WI) and verified by sequencing. Plasmids containing the correct insert (pET30a-HoblOBPs) were then transformed into *E.coli* BL21 (DE3) pLysS competent cells. A single clone of pET30a-HoblOBPs was identified by PCR and sequencing. 

In addition to pET30a-HoblOBP3 and pET30a-HoblOBP4, we also selected four other recombinant plasmids (curated in our lab) including pET30a-HoblOBP1, pET30a-HoblOBP2, pET30a-HoblCSP1 and pET30a-HoblCSP2. All six recombinant plasmids were transformed into *E. coli* BL21 (DE3) pLysS cells and induced with isopropyl-beta-D-thiogalacto-pyranoside (IPTG) at a final concentration of 0.7 mM at 28°C for 8 hours. The samples were sonicated and centrifuged at a low temperature, and the supernatant and pellet were analyzed using sodium dodecyl sulfate polyacrylamide gel electrophoresis (SDS-PAGE). HoblOBP3 and HoblOBP4 were found as inclusion bodies, while the other plasmids were expressed in the supernatant. Soluble proteins were purified using Ni ion affinity chromatography (GE-Healthcare Biosciences, Uppsala, Sweden) for His-tagged-protein purification and anion exchange chromatography (GE Healthcare Biosciences, Uppsala, Sweden). The inclusion body proteins were purified under denaturing conditions (dissolved in 6 M guanidinium hydrochloride buffer) according to previously described redox protocols [56]. Recombinant enterokinase (rEK) (Bio Basic Inc.) was used to remove the His-tag. A second round of Ni ion affinity chromatography was performed to obtain the purified proteins. Next, the proteins were concentrated using Amicon Ultra concentrators with a 10 kDa cutoff (Millipore) and confirmed using SDS-PAGE analysis. The concentrations of the six proteins were then measured using the Bradford method with BSA as the standard protein [75].

### 6 Western blotting analysis

After protein electrophoresis under denaturing conditions (15% SDS-PAGE gel), duplicate gels were prepared for Analysis. 1 gel was stained with 0.1% Coomassie brilliant blue R-250 (in 10% acetic acid, 45% methanol), while the other gel was electrophoresed onto a piece of nitrocellu-lose membrane (Millipore, USA) [5]. After electrophoresis, the membrane was incubated with 5% powdered skimmed milk (0.05% Tween 20 in TBS) overnight. Next, monoclonal mouse anti-His tag fusion protein (California Bioscience, USA) at a dilution of 1:1000 (2 hours) and goat anti-mouse IgG with an alkaline phosphatase labeled (Sigma Aldrich, USA) at a dilution of 1:1000 (1 hour) were incubated sequentially. Immunoreactive bands were detected using 5-bromo-4-chloro-3-indolyl phosphate (BCIP, 0.15 mg/ml) and nitrotetrazolium blue chloride (NBT, 0.3 mg/ml) at a ratio of 1:2.

### 7 Preparation of the antisera

Antisera were obtained by subcutaneously injecting an adult mouse with 50 µg of recombinant HoblOBP3 or HoblOBP4 protein, followed by 3 additional injections of 25 µg on the 21st, 35th, and 49th day. Four mice were used in a parallel study. The proteins were emulsiﬁed with an equal volume of Freund’s complete adjuvant on the ﬁrst injection and Freund’s incomplete adjuvant on the second injection. The antiserum was then tested using an enzyme-linked immunosorbent assay. The mice were exsanguinated 10 days after the last injection, and the serum was used without further puriﬁcation. Other HoblOBPs antisera were obtained by injecting adult rabbits.

### 8 Ethic statement

I proclaimed that the following statement was accurate. All animal procedure in this study was strictly performed according to guidelines developed by the ethics committee of the State Key Laboratory for Biology of Plant Diseases and Insect Pests, Institute of Plant Protection, Chinese Academy of Agricultural Sciences. The approval ID or permit numbers is SYXK (Beijing) 2008-008. All animal procedure was performed under anesthesia, and wounds were cleaned that before they got infected. All efforts were made to minimize suffering.

### 9 Fluorescence binding assays

N-phenyl-1-naphthylamine (1-NPN) was selected as a probe to measure the affinity of the 1-NPN fluorescent ligand to proteins [5,13,14]. A 1-cm light path quartz cuvette was used, and the fluorescence spectra were recorded on a Lengguang 970 CRT spectrofluorimeter (Shanghai Jingmi, China) at room temperature in a right angle configuration. The parameter selection was such that the slit widths for both excitation and emission were 10 nm. 1-NPN was excited at 337 nm and the emission spectra were recorded between 350 and 550 nm. Spectra were recorded using high-speed scanning. Several types of compounds purchased from Sigma-Aldrich (Chemie Gmbh, Steinheim, Germany) were identified in the binding assays, and their purities were > 97% (Table 1).

The 2 µM protein solution was dissolved in 50 mM Tris-HCl buffer with pH 7.4, and the ligands were dissolved in chromatographically pure methanol as a 1 mM stock solution. 1-NPN was dissolved in chromatographically pure methanol as a 1 mM stock solution. The protein solution was titrated to measure the protein’s affinity for the probe by adding aliquots of 1-NPN stock solution to final concentrations of 2 to 20µM. The affinity of the ligands was estimated using competitive binding assays with both 1-NPN and proteins at 2 µM; the final concentrations for each competitive ligand were in the range of 2 to 24 µM.

To determine the dissociation constants, the intensity values corresponding to the maximum fluorescence emission were plotted against free ligand concentrations. Assuming that the protein was 100% active and that the stoichiometric ratio between the protein and ligand was 1:1 at saturation, the bound ligand was determined from the fluorescence intensity values. The curves were then linearized using Scatchard plots. The K_1-NPN_ values were estimated using GraphPad Prism 5 Software by nonlinear regression for a unique binding site [49,76-78]. The dissociation constants of the competitors (*Ki*) were calculated from the corresponding IC50 values using the following equation: Ki = [IC50]/1+[1-NPN]/K_1-NPN_. [IC50] was defined as the concentration of a competitor that caused a 50% reduction in the fluorescence intensity. [1-NPN] represented the free concentration of 1-NPN, and K_1-NPN_ represented the dissociation constant of the complex protein/1-NPN [76].

### 10 Colocalization of transmission electron microscopy (TEM) and immunocytochemistry

The antennae lemalla of adult beetles were excised and chemically ﬁxed in a mixture of paraformaldehyde (4%) and glutaraldehyde (2%) in 0.1 M phosphate-buffered saline (pH 7.4). After dehydration in an ethanol series, the samples were embedded in LR White resin (Taab, Aldermaston, Berks, UK). Ultrathin sections were cut using a diamond knife and initially treated with primary antisera against HoblOBPs, which were diluted at 1:3000–1:10000. The secondary antibody was anti-mouse IgG conjugated to 5-nm colloidal gold or anti-rabbit IgG conjugated to 10-nm colloidal gold (Sigma, St. Louis, MO), which was diluted to a ratio of 1:20. Gold granules were amplified using silver-intensification. Next, the sections were stained with 2% uranyl acetate to increase the contrast for transmission electron microscopy analysis (HITACHIH-7500) [63,68,79,80]. 

Double-labelling for HoblOBP1 and HoblOBP2 or HoblOBP2 and HoblOBP4 was performed using both antisera on the same grid, according to a previously described method [80]. For HoblOBP2 and HoblOBP4 double-labelling, the primary HoblOBP2 antibodies were incubated for 120 min at room temperature and subsequently incubated with goat-anti-rabbit IgG conjugated with 10-nm gold, as described above. Silver enhancement was then performed. Next, the sections were labeled using HoblOBP4 antibodies and goat-anti-mouse lgG conjugated with 5-nm gold without silver enhancement. Then, 2% uranyl acetate was used to stain the sections. The two labels could be easily discriminated from each other. This double-labelling method for HoblOBP1 and HoblOBP2 was performed with silver enhancement again after the second labeling.
